# Single nucleotide polymorphisms in DNA repair genes might be prognostic factors in muscle-invasive bladder cancer patients treated with chemoradiotherapy

**DOI:** 10.1038/sj.bjc.6603290

**Published:** 2006-08-01

**Authors:** S Sakano, T Wada, H Matsumoto, S Sugiyama, R Inoue, S Eguchi, H Ito, C Ohmi, H Matsuyama, K Naito

**Affiliations:** 1 Department of Urology, Yamaguchi University School of Medicine, 1-1-1 Minami-Kogushi, Ube, Yamaguchi 755-8505, Japan; 2 Department of Public Health, Yamaguchi University School of Medicine, 1-1-1 Minami-Kogushi, Ube, Yamaguchi 755-8505, Japan

**Keywords:** single nucleotide polymorphism, DNA repair gene, muscle-invasive bladder cancer, chemoradiotherapy, survival

## Abstract

DNA repair enzymes repair DNA damaged by platinum agents and ionising radiation. Single nucleotide polymorphisms (SNPs) in DNA repair genes modulate the repair capacity and might affect response and prognosis following platinum-based chemoradiotherapy (CRT). We investigated associations between the functional SNPs in DNA repair genes and response and survival in muscle-invasive bladder cancer patients treated with CRT to determine the predictive value of the SNPs in patient selection for bladder conservation therapy. The study group comprised 78 patients who underwent CRT for transitional cell carcinoma of the bladder. Single nucleotide polymorphisms in xeroderma pigmentosum complementation groups C (Lys939Gln, A/C), D (XPD; Lys751Gln, A/C), and G (Asp1104His, G/C), and X-ray repair cross-complementing groups 1 (XRCC1; Arg399Gln, G/A) and 3 (Thr241Met, T/C) genes were genotyped. Combined genotypes with at least one variant allele in XPD or XRCC1 were significantly associated with improved cancer-specific survival compared with remaining groups (*P*=0.009). In multivariate analysis, only the combined XPD and XRCC1 genotypes were independently associated with cancer-specific survival (*P*=0.04). The association was stronger in stage T3/T4 patients (*P*=0.0008). These results suggest that combined XPD and XRCC1 genotypes might be prognostic factors in muscle-invasive bladder cancer patients treated with CRT.

The complex system of DNA repair enzymes plays a vital role in protecting the genome from the consequences of mutagenic exposure (Hoeijmakers, 2001). At least four pathways of DNA repair: base excision repair (BER), nucleotide excision repair (NER), double-strand-break (DSB) repair and mismatch repair, operate on specific types of damaged DNA and each involves numerous molecules (Goode *et al*, 2002). There are several common single nucleotide polymorphisms (SNPs) in genes encoding DNA repair enzymes and many case–control studies have examined the relationship between SNPs and susceptibility to different types of cancer (Goode *et al*, 2002). Some of the SNPs in DNA repair genes reportedly result in a subtle structural alteration of the repair enzyme and modulation of the repair capacity (Lunn *et al*, 1999; Spitz *et al*, 2001; Matullo *et al*, 2003; Vodicka *et al*, 2004).

NER is an essential pathway of DNA repair and the only known mechanism in mammalian cells for the removal of bulky, helix-distorting DNA adducts produced by platinum agents (Park *et al*, 2001). Moreover, DNA damaged by ionising radiation is repaired mainly by the BER pathway and by homologous recombination repair (HRR) and nonhomologous end-joining repair, which are types of DSB repair (Hu *et al*, 2001; Khanna and Jackson, 2001). Modulation of repair capacity by the SNPs in DNA repair genes might therefore affect response and prognosis following platinum-based chemoradiotherapy (CRT) for cancer. The associations between SNPs and clinical response or prognosis have been reported in patients with lung or colorectal cancer treated with platinum-based chemotherapy or radiotherapy (Park *et al*, 2001; Gurubhagavatula *et al*, 2004; Ryu *et al*, 2004; Stoehlmacher *et al*, 2004; Zhou *et al*, 2004; Yoon *et al*, 2005).

Although the gold standard for the treatment of muscle-invasive urinary-bladder cancer is radical cystectomy followed by urinary diversion, this procedure is likely to impair the quality of life of the patient (Rödel *et al*, 2002). In many areas of cancer treatment, the trend in the 1990s was aimed at organ conservation using combined chemotherapy and radiation with or without conservative local surgery (Shipley *et al*, 2003). In invasive bladder cancer, several groups have reported the value of combined-modality therapy, including transurethral resection (TUR), radiation therapy, and platinum-based systemic chemotherapy (Rödel *et al*, 2002; Shipley *et al*, 1998, 2003). With these programs, cystectomy is reserved for patients with an incomplete response or local relapse after combined-modality treatment. Five-year survival rates in the range of 50–65% have been published in these series, and approximately three quarters of the surviving patients maintained their own bladders (Tester *et al*, 1996; Kachnic *et al*, 1997; Dunst *et al*, 2001). However, combined-modality therapy may not only be potentially harmful but also diminish survival as a result of the delay in cystectomy for some patients with nonresponding bladder tumours. Thus, predictors of response and prognosis after therapy that help determine appropriate patients for bladder preservation are sorely required.

In the current study, we investigated the associations between functional SNPs in DNA repair genes (xeroderma pigmentosum complementation groups C (XPC), D (XPD), and G (XPG) (involved in NER), and X-ray repair cross-complementing groups 1 (XRCC1) (involved in BER), and 3 (XRCC3) (involved in HRR)) and the clinical response and survival in patients with locally invasive bladder cancer treated with platinum-based CRT to determine the predictive value of the SNPs in patient selection for bladder conservation therapy.

## MATERIALS AND METHODS

### Patients

The ethical review committee of Yamaguchi University School of Medicine approved this study. The study group comprised 78 patients who underwent CRT for locally muscle-invasive (T2-4N0M0) or high-risk superficial (T1G3) urinary-bladder cancer at the Department of Urology, Yamaguchi University School of Medicine, Ube, Japan, from January 1995 to October 2004. The median age was 68 years (range, 29–89 years) and patients included 59 men (75.6%) and 19 women (24.4%). Before treatment, all patients underwent tumoural and random-mucosal biopsies of the bladder transurethrally and a computerised tomography (CT) scan for staging. Patients were staged according to the TNM staging system of the UICC (1992): five (6.4%) were stage T1G3, 35 (44.9%) stage T2, 30 (38.5%) stage T3, and eight (10.3%) stage T4. All bladder tumours were histopathologically confirmed as transitional cell carcinoma (TCC). Of the 78 tumours, 69 showed TCC only, five showed an adenocarcinoma component, and four showed squamous differentiation. The tumours were graded according to the WHO classification: the majority (*n*=60, 76.9%) were grade 3 and the remaining 18 (23.1%) were grade 2.

### CRT

All patients received combined platinum-based systemic chemotherapy and radiation therapy. In most patients, the regimen (based on Shipley's method with slight modification) (Shipley *et al*, 1987) for one cycle was cisplatin (70 mg m^−2^) on day 1, with radiation administered by Liniac to the true pelvis at 1.8 grays (Gy) per fraction from day 2 to day 5 in the first week, and on five consecutive days in the second week (Matsumoto *et al*, 2004a). The photon energy of the Liniac was 10 MV and patient-specific target volume was defined by CT planning. This therapy was carried out one to three times every 14 days. At completion of one cycle of CRT, patients were treated with 70 mg m^−2^ cisplatin and irradiation of the basic target at 16.2 Gy. Although we tried to perform three cycles of CRT where possible, the treatment was halted in patients who received one or two cycles of therapy and showed persistent side effects such as nausea, vomiting, diarrhea, or pancytopenia for 2 weeks or who refused to continue with CRT. The median total doses of cisplatin and radiation were 235 mg (range, 120–400 mg) and 48.6 Gy (range, 30–60.4 Gy), respectively.

At 4 weeks after the completion of CRT, patients were assessed for response by random-mucosal biopsy or TUR and a CT scan. Patients with nonresponding tumours were referred for radical or partial cystectomy: salvage cystectomy was undertaken in 17 patients (21.8%). Patients with responding tumours underwent complete resection of the cancer by TUR. A complete response (CR) was defined as no residual tumour detected pathologically, a partial response (PR) as a residual nonmuscle-invasive tumour, and no change (NC) as a residual muscle-invasive tumour. Complete response was observed in 35.9% (28 of 78), PR in 43.6% (34 of 78), and NC in 20.5% (16 of 78). Twelve patients with residual carcinoma *in situ* received intravesical instillation of BCG. Cystoscopic examination followed by washing cytology was carried out every 3 months for the first 5 years and every 6 months thereafter. Complementary examinations, including chest X-rays and/or CT scans, were carried out every 6 months. The median duration of follow-up was 45 months (range, 2–124 months). Of the 67 patients who achieved a state of no evidence of disease after combined-modality therapy, 37 (55.2%) had superficial or invasive-local recurrence or distant metastasis. A total of 17 patients suffered cancer-related death during the follow-up.

### DNA extraction and genotyping

Venous blood samples were collected from each patient before tumour biopsies. Lymphocyte DNA was extracted using the QIAamp DNA Mini Kit (VWR International, West Chester, PA, USA) or GenTLE extraction kit (Takara, Tokyo, Japan). Single nucleotide polymorphisms in XPC (Lys939Gln, A/C), XPD (Lys751Gln, A/C), XPG (Asp1104His, G/C), XRCC1 (Arg399Gln, G/A), and XRCC3 (Thr241Met, T/C) genes were genotyped using polymerase chain reaction–restriction fragment length polymorphism (PCR–RFLP). The method, including primer sequences and restriction endonucleases for each SNP, was described previously (Sanyal *et al*, 2004; Sakano *et al*, 2006). Briefly, the DNA fragments were amplified from 10 ng of DNA in 10 *μ*l PCR reactions containing 1.5 mM MgCl_2_, 0.2 mM each dNTP, 0.3 U AmpliTaq Gold DNA polymerase (Applied Biosystems, Foster City, CA, USA) and 0.3 *μ*M of each primer. The PCR products were digested with the appropriate restriction endonucleases that recognised and cut either wild-type or variant sequences at 37°C for at least 3 h. The digested PCR products were electrophoresed on 2% agarose gels and stained with ethidium bromide for visualisation under UV light. In order to control the restriction digestion of the PCR products, genotyping assays were randomly repeated and results were checked for concordance. About 10% of the amplified fragments were also randomly checked by direct DNA sequencing and comparison of the PCR–RFLP and sequencing results showed 100% concordance in all experiments (Kumar *et al*, 2003; Sakano *et al*, 2003).

### Statistical analysis

Relationships between genotypes of the DNA repair genes and tumour stage, grade, response to CRT, and recurrence were assessed using the *χ*^2^-test (with Yates' correction) and the odds ratio (OR) or risk ratio (RR) with 95% confidence intervals (CI) were calculated. Bonferroni correction was taken into consideration because multiple comparisons were carried out. For this correction, a *P*-value of ⩽0.006 was considered statistically significant. The outcome selected for this study was cancer-specific survival, defined as the time from the start of CRT to the date of death from muscle-invasive bladder cancer. Cancer-specific survival was analysed by plotting Kaplan–Meier curves and the survival probability distributions were compared using the log-rank test. Categorical variables influencing survival were compared using Cox proportional hazards regression. Data were processed using JMP software (SAS Institute Inc., Cary, NC, USA), with *P*<0.05 indicating statistical significance. Variables with *P*<0.05 in univariate analysis were also assessed for their relationship with cancer-specific survival in multivariate analysis.

## RESULTS

### Genotypes of DNA repair genes and clinical prognostic features

Genotype frequencies of SNPs in XPC, XPD, XPG, XRCC1, and XRCC3 genes were found to be in Hardy–Weinberg equilibrium. The variant allele frequencies for the SNPs were 44.4, 6.9, 59.1, 22.2, and 6.5%, respectively. The numbers of variant alleles for XPD and XRCC1 were not associated with one another (*P*=0.91; *χ*^2^-test; Table 1). No associations were detected between the genotypes and age or gender. None of the genotypes were associated with histopathology of the tumours (TCC only, TCC with adenocarcinoma element, or TCC with squamous cell carcinoma element). None of the evaluated genotypes were associated with tumour stage or grade (*χ*^2^-test (with Yates' correction); Table 2). The relationships between the genotypes of DNA repair genes and response to CRT and tumour recurrence or metastasis are presented in Table 3. The genotypes were not associated with response to CRT at 4 weeks after evaluation. There were no relationships between the genotypes of XPC, XPD, XPG, XRCC1, XRCC3, or combined XPD and XRCC1 genes and recurrence during follow-up. The recurrence rate was significantly lower in patients with greater numbers of total variant alleles both in all DNA repair genes studied and in NER genes: XPC, XPD, and XPG (>3 compared with ⩽3, RR: 0.70, 95% CI: 0.49–0.99, *P*=0.03; >2 compared with ⩽2, RR: 0.66, 95% CI: 0.44–0.99, *P*=0.03; respectively; *χ*^2^-test). However, when the Bonferroni correction for multiple comparisons was taken into consideration, the recurrence rate was not significantly lower in those patients.

### Genotypes of DNA repair genes and cancer-specific survival

**Figure 1:**
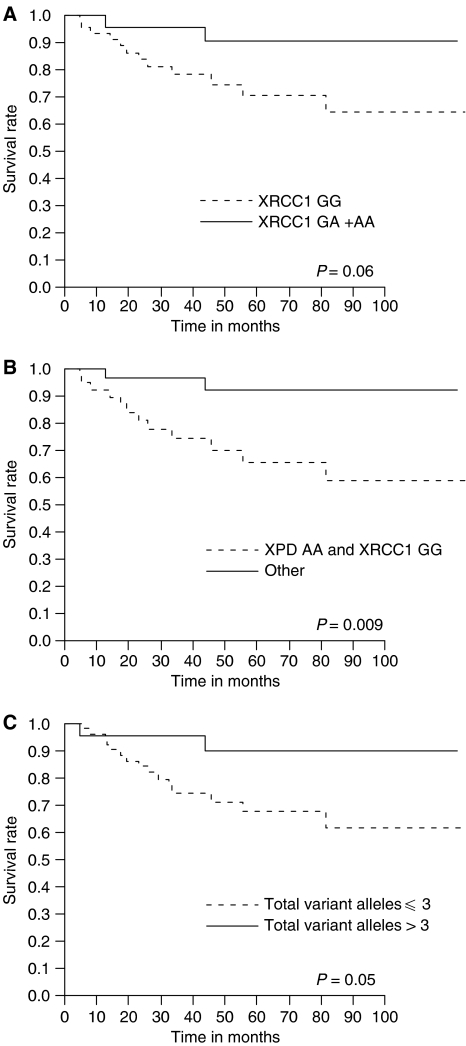
Kaplan–Meier cancer-specific survival curves for all muscle-invasive bladder cancer patients treated with chemoradiotherapy. (**A**) XRCC1 GG *vs* GA+AA genotypes; (**B**) patients stratified by combined XPD and XRCC1 genotypes: XPD AA and XRCC1 GG *vs* other genotypes; (**C**) patients stratified by the number of total variant alleles in DNA repair genes: ⩽3 *vs* >3.

In univariate analysis using the Cox proportional hazards regression model, XRCC1 genotypes, combined XPD and XRCC1 genotypes, and the number of total variant alleles in DNA repair genes were significantly associated with cancer-specific survival (RR: 0.51, 95% CI: 0.20–0.98, *P*=0.04 for XRCC1; RR: 0.41, 95% CI: 0.16–0.79, *P*=0.006 for combined XPD and XRCC1; RR: 0.51, 95% CI: 0.20–0.95, *P*=0.03 for variant alleles; Table 4), so patients with XRCC1 GA+AA genotypes, with at least one variant allele in XPD or XRCC1, and with more than 3 variant alleles in DNA repair genes had improved cancer-specific survival. Cancer-specific survival was plotted for the above-mentioned genotypes using Kaplan–Meier survival curves (Figure 1A–C, respectively). Combined genotypes with at least one variant allele in XPD or XRCC1 were significantly associated with improved cancer-specific survival compared with remaining groups (*P*=0.009; log-rank test). However, there were no significant differences with regard to the XRCC1 genotypes or number of variant alleles in DNA repair genes (*P*=0.06 and *P*=0.05, respectively; log-rank test).

Clinical variables were also assessed for their relationship with cancer-specific survival (Table 4). In univariate analysis, tumours with adenocarcinoma or squamous cell carcinoma elements, high-dose radiation (⩾48.6 Gy), and a nonresponse to CRT (NC) were significantly associated with an unfavourable outcome (RR: 2.15, 95% CI: 1.13–3.65, *P*=0.02 for other elements; RR: 2.56, 95% CI: 1.45–5.36, *P*=0.0007 for radiation dose; RR: 1.75, 95% CI: 1.01–2.89, *P*=0.04 for response to CRT). In multivariate analysis (model one) including all significant factors (other elements, radiation dose, response to CRT, XRCC1 genotypes, and number of total variant alleles in DNA repair genes), only high-dose radiation was significantly associated with an unfavourable outcome (RR: 2.00, 95% CI: 1.06–4.35, *P*=0.03). When combined XPD and XRCC1 genotypes were used instead of XRCC1 genotypes and the number of variant alleles in the DNA repair genes in multivariate analysis (model two), only the combined XPD and XRCC1 genotypes were independently associated with cancer-specific survival (RR: 0.50, 95% CI: 0.19–0.97, *P*=0.04).

**Figure 2:**
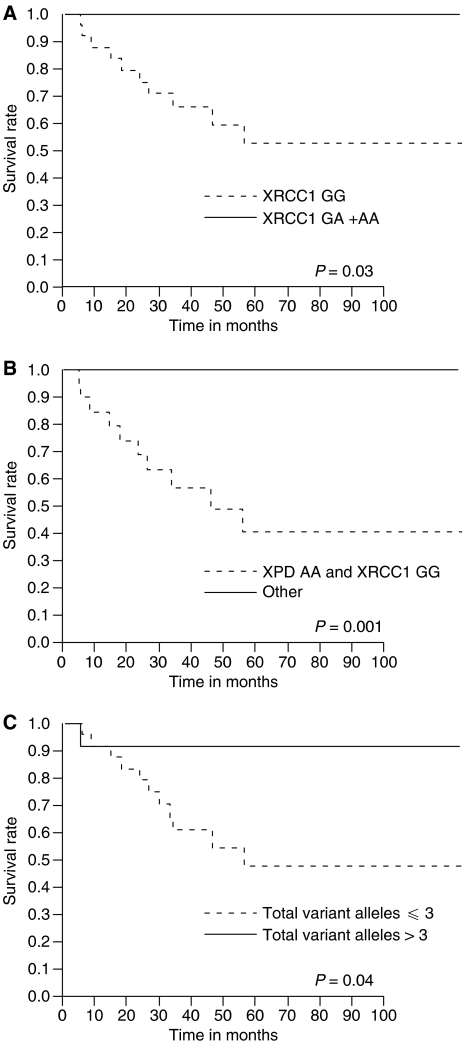
Kaplan–Meier cancer-specific survival curves for stage T3/T4 bladder cancer patients treated with chemoradiotherapy. (**A**) XRCC1 GG *vs* GA+AA genotypes; (**B**) patients stratified by combined XPD and XRCC1 genotypes: XPD AA and XRCC1 GG *vs* other genotypes; (**C**) patients stratified by the number of total variant alleles in DNA repair genes: ⩽3 *vs* >3.

In stage T3/T4 patients, cancer-specific survival was plotted for XRCC1 genotypes, combined XPD and XRCC1 genotypes, and the number of variant alleles in DNA repair genes (Figure 2A–C, respectively). All were significantly associated with cancer-specific survival (*P*=0.03, *P*=0.001, and *P*=0.04, respectively; log-rank test). In multivariate analysis, the XRCC1 genotypes (model one) and the combined XPD and XRCC1 genotypes (model two) were independently associated with cancer-specific survival in stage T3/T4 patients (RR: 0.0004, 95% CI: not calculated, *P*=0.02; RR: 0.0003, 95% CI: 0.00–0.53, *P*=0.0008, respectively; Table 5).

## DISCUSSION

Previous reports have revealed significant associations between XPD or XRCC1 gene polymorphisms and survival of lung or colorectal cancer patients treated with platinum-based chemotherapy or radiotherapy (Park *et al*, 2001; Gurubhagavatula *et al*, 2004; Stoehlmacher *et al*, 2004; Yoon *et al*, 2005). Concerning bladder cancer, Gu *et al* (2005) showed that the NER gene polymorphisms have significant impacts on recurrence and recurrence-free survival time in superficial cancer. Moreover, Sak *et al* (2005) indicated that high expression levels of APE1 and XRCC1 are associated with improved cancer-specific survival following radical radiotherapy in muscle-invasive bladder cancer. However, reports on the associations between DNA repair gene polymorphisms and clinical response or prognosis in muscle-invasive bladder cancer patients treated with chemotherapy or radiotherapy are lacking.

We found a significant relationship between combined XPD and XRCC1 genotypes and cancer-specific survival in muscle-invasive bladder cancer patients treated with CRT. We also analysed stage T3/T4 patients to assess the findings in confirmedly advanced bladder tumours locally, because the staging on CT scan has limited accuracy in some bladder cancer patients (Paik *et al*, 2000). The relationship between the combined XPD and XRCC1 genotypes and cancer-specific survival was stronger in stage T3/T4 patients. The combined genotypes might therefore be valuable as an independent prognostic factor following CRT in muscle-invasive bladder cancer. Bladder-conserving therapy using combinations of chemotherapy, radiotherapy, and TUR has shown promising results (Dunst *et al*, 2001; Rödel *et al*, 2002), but not all tumours respond. The ability to predict tumour sensitivity to CRT would therefore be very helpful in patient selection for bladder conservation therapy (Sak *et al*, 2005). In addition, most of the molecular markers assessed for prognostic potential in bladder cancer need tumour tissue samples and complex techniques (Garcia del Muro *et al*, 2004; Matsumoto *et al*, 2004a, 2004b; Chakravarti *et al*, 2005), unlike the evaluation of germline genetic polymorphisms, which requires little more than a peripheral blood sample and standard PCR-based reactions, making these tests potentially useful in the clinical setting (Gurubhagavatula *et al*, 2004).

In our series, the median dose of radiotherapy at 48.6 Gy was low compared with radical radiotherapy for bladder cancer. However, Dunst *et al* (2001) stated that from radiobiological considerations, 50 Gy radiation should be able to control disease in combination with complete transurethral surgery in the vast majority of patients, and that this dose can be safely administered in the pelvis, as is known from treatments of other pelvic malignancies. They also indicated that the addition of (mainly cisplatin based) chemotherapy improves local complete remission rates irrespective of the radiation dose (Dunst *et al*, 2001).

Platinum compounds such as cisplatin form both intra- and interstrand DNA adducts that result in bulky distortion of DNA, destabilisation of the double helix, and inhibition of DNA replication (Gurubhagavatula *et al*, 2004). These adducts are responsible for the cytotoxicity of the drug, and clinical outcome seems to be correlated with the level of platinum-DNA adducts in the circulation (Reed *et al*, 1987; Bosken *et al*, 2002). Additionally, these adducts are mainly repaired by the NER pathway (Park *et al*, 2001). One hypothesis regarding DNA repair capacity and muscle-invasive bladder cancer outcome is therefore that suboptimal DNA repair within the tumour might actually lead to decreased removal of platinum-DNA adducts, and thus, increased clinical response and survival after platinum therapy (Bosken *et al*, 2002).

Ionising radiation exposure damages cellular DNA in many ways, requiring the concerted action of a number of DNA repair enzymes to restore genomic integrity (Hu *et al*, 2001). The BER and HRR pathways are particularly important with regard to this (Thompson and West, 2000; Khanna and Jackson, 2001). As oxidative base damage and strand breaks induced by ionising radiation are repaired mainly by these two pathways, defective repair activities might contribute to sensitivity to and improved survival following radiotherapy. Thus, we selected DNA repair genes: XPC, XPD, and XPG (involved in NER), XRCC1 (involved in BER), and XRCC3 (involved in HRR), as the focus of this study. Furthermore, the SNPs that we studied in these genes are thought to be associated with different levels of DNA repair activity (Lunn *et al*, 1999; Spitz *et al*, 2001; Matullo *et al*, 2003; Vodicka *et al*, 2004), and might explain interindividual differences in response and prognosis after CRT for muscle-invasive bladder cancer. We also analysed relationships between the combined XPD and XRCC1 genotypes and clinical factors. As both XPD and XRCC1 genes are located close to each other at 19q13.2–13.3 (Mohrenweiser *et al*, 1989), analysis of the combined XPD and XRCC1 genotypes might be meaningful with regard to specific haplotypes.

Yoon *et al* (2005) showed that the variant-type XRCC1 Arg399Gln genotype is associated with progression-free survival advantage in non–small-cell lung cancer patients who have received radiotherapy. Moreover, Hu *et al* (2001) indicated that amino-acid substitution variants of XRCC1 and APE1 might contribute to ionising radiation hypersensitivity. These results are in accord with our findings that the wild-type genotypes of both XPD and XRCC1 are associated with shorter cancer-specific survival in muscle-invasive bladder cancer patients treated with CRT. However, the results of Hu *et al* (2001) seem to contradict those of Sak *et al* (2005) noted above. Sak *et al* (2005) stated that one possible explanation is that the reduced expression of XRCC1 and APE1 merely reflects the poorly differentiated nature of tumour cells in more aggressive tumours, and that cells from aggressive tumours with extensive genomic instability could contain chromosomal aberrations that result in the failure of transcription of genes, including DNA repair genes, thus resulting in lower protein expression of the gene products.

Gu *et al* (2005) analysed the association between the total number of variant alleles in eight NER genes and the recurrence of superficial bladder cancer. They emphasised that because the prognosis of cancer patients likely involves multistep, multigenic pathways, it is unlikely that any one single genetic polymorphism would have a dramatic effect on clinical outcome, and that it is important to take a pathway-based analysis of multiple polymorphic genes (Gu *et al*, 2005). Thus, we also analysed the numbers of total variant alleles both in all DNA repair genes studied and in NER genes. Although the numbers of total variant alleles were significantly associated with recurrence and cancer-specific survival (univariate analysis), no significant relationships were found between specific combinations of variant alleles and recurrence or cancer-specific survival (data not shown). The number of total variant alleles was not significantly associated with either recurrence after application of Bonferroni correction or cancer-specific survival in multivariate analysis.

Gurubhagavatula *et al* (2004) showed that increasing numbers of either XPD or XRCC1 variant alleles and a greater number of variant alleles in combinations of both genes are associated with shorter overall survival after therapy with platinum agents for non-small-cell lung cancer. Further, Park *et al* (2001) indicated that metastatic colorectal cancer patients with the wild-type XPD genotype respond to 5-fluorouracil/oxaliplatin and have longer median survival *vs* those with the variant genotypes. These results are not consistent with our findings. This discrepancy may depend on cancer type or therapies combined with radiation. Cisplatin has been shown to enhance the cytotoxicity of radiation in both cell culture and tumour-bearing animals, although the mechanisms of the interaction have not been defined (Schilsky, 1992). DNA repair enzymes might decrease the synergistic effects of combination of cisplatin and radiation. The reason for the discrepancy could be clarified by investigating the relationship between the genotypes of DNA repair genes and outcomes in bladder cancer patients treated with other modalities such as platinum-based systemic chemotherapy alone and radiotherapy alone. Additionally, to remove gene prevalence as a confounding event, validation with an independent sample set is needed, as is a sample set from a different ethnic background.

In conclusion, combined XPD and XRCC1 genotypes were shown to be associated with cancer-specific survival in muscle-invasive bladder cancer patients treated with CRT. The results suggest that these genotypes might be prognostic factors following CRT in bladder cancer, helping patient selection for bladder conservation therapy. However, with the limited sample size, our results allow only preliminary conclusions, but warrant larger studies to further test the prognostic significance of the genotypes of DNA repair genes in muscle-invasive bladder cancer patients treated with CRT.

## Figures and Tables

**Table 1:** Distribution of XPD and XRCC1 genotypes

	**XRCC1 genotype (Arg399Gln, G/A)**		
**XPD genotype (Lys751Gln, A/C)**	**GG**	**GA**	**AA**
AA	40	16	5
AC	7	2	1
CC	0	0	0

*P*-value=0.91.

**Table 2:** Relationship between genotypes of DNA repair genes and tumour stage and grade

	**Stage**				**Grade**			
**Genotype**	**T1G3/T2**	**T3/T4**	**Odds ratio (95% CI)**	***P*-value**	**2**	**3**	**Odds ratio (95% CI)**	***P*-value**
*XPC (Lys939Gln, A/C)*								
AA	11	10			5	16		
AC	19	18	1.04 (0.36–3.04)	0.94	8	29	1.13 (0.32–4.05)	0.89
CC	7	6	0.94 (0.24–3.77)	0.79	3	10	1.04 (0.20–5.34)	0.71
AC+CC	26	24	1.02 (0.37–2.82)	0.98	11	39	1.11 (0.33–3.70)	0.88
								
*XPD (Lys751Gln, A/C)* a								
AA	35	27			15	47		
AC+CC	2	8	5.19 (1.02–26.43)	0.07	2	8	1.28 (0.24–6.68)	0.91
								
*XPG (Asp1104His, G/C)*								
GG	6	6			3	9		
GC	18	21	1.17 (0.32–4.26)	0.92	10	29	0.97 (0.22–4.30)	0.74
CC	15	11	0.73 (0.19–2.90)	0.93	5	21	1.40 (0.27–7.15)	0.98
GC+CC	33	32	0.97 (0.28–3.32)	0.79	15	50	1.11 (0.27–4.64)	0.82
								
*XRCC1 (Arg399Gln, G/A)* a								
GG	21	26			12	35		
GA+AA	16	9	0.45 (0.17–1.23)	0.12	5	20	1.37 (0.42–4.46)	0.60
								
*XRCC3 (Thr241Met, C/T)* a								
CC	31	29			14	46		
CT+TT	5	4	0.86 (0.21–3.50)	0.89	2	7	1.07 (0.20–5.72)	0.73
								
*Combined XPD and XRCC1*								
XPD AA and XRCC1 GG	20	20			10	30		
Other	17	15	0.88 (0.35–2.24)	0.79	7	25	1.19 (0.40–3.58)	0.76
								
*Total variant alleles in DNA repair genes*								
⩽3	29	26			13	42		
>3	11	12	1.22 (0.46–3.22)	0.69	5	18	1.11 (0.35–3.59)	0.86
								
*Total variant alleles in NER genes* b								
⩽2	28	22			12	38		
>2	12	16	1.70 (0.67–4.32)	0.27	6	22	1.16 (0.38–3.52)	0.80

NER=nucleotide excision repair.

a For those polymorphisms with few homozygous variant alleles, only the combined results of the heterozygous and homozygous variant alleles are shown.

b NER genes include XPC, XPD, and XPG.

**Table 3:** Relationship between genotypes of DNA repair genes and response to chemoradiotherapy and recurrence or metastasis

	**Response**				**Recurrence**			
**Genotype**	**CR/PR**	**NC**	**Risk ratio (95% CI)**	***P*-value**	**No**	**Yes**	**Risk ratio (95% CI)**	***P*-value**
*XPC (Lys939Gln, A/C)*								
AA	16	5			7	13		
AC	27	10	1.12 (0.49–2.52)	0.79	16	14	0.63 (0.30–1.31)	0.20
CC	12	1	0.69 (0.42–1.11)	0.46	7	5	0.69 (0.38–1.26)	0.36
AC+CC	39	11	0.93 (0.40–2.15)	0.87	23	19	0.57 (0.27–1.24)	0.15
								
*XPD (Lys751Gln, A/C)* a								
AA	47	15			24	30		
AC+CC	9	1	0.90 (0.75–1.06)	0.55	6	3	0.88 (0.71–1.08)	0.38
								
*XPG (Asp1104His, G/C)*								
GG	9	3			5	5		
GC	34	5	0.56 (0.19–1.62)	0.58	16	17	1.05 (0.35–3.10)	0.78
CC	18	8	1.22 (0.41–3.68)	0.98	9	14	1.36 (0.49–3.80)	0.84
GC+CC	52	13	0.79 (0.24–2.57)	>0.99	25	31	1.20 (0.38–3.76)	0.97
								
*XRCC1 (Arg399Gln, G/A)* a								
GG	35	12			20	20		
GA+AA	21	4	0.83 (0.59–1.18)	0.53	10	12	1.07 (0.74–1.54)	0.73
								
*XRCC3 (Thr241Met, C/T)* a								
CC	46	14			25	28		
CT+TT	8	1	0.91 (0.77–1.09)	0.69	5	2	0.89 (0.74–1.08)	0.42
								
*Combined XPD and XRCC1*								
XPD AA and XRCC1 GG	29	11			16	18		
Other	27	5	0.75 (0.50–1.14)	0.23	14	14	0.95 (0.60–1.49)	0.82
								
*Total variant alleles in DNA repair genes*								
⩽3	44	11			17	30		
>3	18	5	1.03 (0.72–1.49)	0.86	13	7	0.70 (0.49–0.99)	0.03^b^
								
*Total variant alleles in NER genes* c								
⩽2	41	9			15	28		
>2	21	7	1.18 (0.74–1.88)	0.66	15	9	0.66 (0.44–0.99)	0.03^b^

CR=complete response; PR=partial response; NC=no change; NER=nucleotide excision repair.

a For those polymorphisms with few homozygous variant alleles, only the combined results of the heterozygous and homozygous variant alleles are shown.

b Differences not significant after application of Bonferroni correction as *P*-values are >0.006.

c NER genes include XPC, XPD, and XPG.

**Table 4:** Univariate and multivariate regression analyses for predicting cancer-specific survival in all bladder cancer patients treated with chemoradiotherapy

	**Univariate model**		**Multivariate model one**		**Multivariate model two**	
**Variable (*n*)**	**Risk ratio (95% CI)**	***P*-value**	**Risk ratio (95% CI)**	***P*-value**	**Risk ratio (95% CI)**	***P*-value**
*Age (years)*						
⩽68 (41)						
>68 (37)	1.31 (0.81–2.15)	0.27				
						
*Gender*						
Men (59)						
Women (19)	0.97 (0.52–1.64)	0.92				
						
*Tumour stage*						
T1G3/T2 (40)						
T3/T4 (38)	1.61 (0.98–2.85)	0.06				
						
*Tumour grade*						
2 (18)						
3 (60)	0.81 (0.50–1.44)	0.45				
						
*Histopathology*						
Pure TCC (69)						
Other elements (9)	2.15 (1.13–3.65)	0.02	1.80 (0.83–3.47)	0.13	1.85 (0.84–3.58)	0.11
						
*Cisplatin dose (mg)*						
<235 (37)						
⩾235 (37)	1.27 (0.78–2.10)	0.34				
						
*Radiation dose (Gy)*						
<48.6 (38)						
⩾48.6 (40)	2.56 (1.45–5.36)	0.0007	2.00 (1.06–4.35)	0.03	1.86 (0.99–4.02)	0.05
						
*Response*						
CR/PR (62)						
NC (16)	1.75 (1.01–2.89)	0.04	1.76 (0.96–3.22)	0.07	1.79 (0.98–3.24)	0.06
						
*Salvage cystectomy*						
No (61)						
Yes (17)	0.81 (0.39–1.42)	0.49				
						
*XPC (Lys939Gln, A/C)*						
AA (21)						
AC (37)	0.66 (0.18–2.38)	0.52				
CC (13)	0.93 (0.19–3.79)	0.92				
AC+CC (50)	0.87 (0.50–1.58)	0.62				
						
*XPD (Lys751Gln, A/C)* a						
AA (62)						
AC+CC (10)	0.68 (0.16–1.53)	0.41				
						
*XPG (Asp1104His, G/C)*						
GG (12)						
GC (39)	1.56 (0.40–10.24)	0.55				
CC (26)	1.50 (0.34–10.21)	0.61				
GC+CC (65)	1.23 (0.65–3.11)	0.56				
						
*XRCC1 (Arg399Gln, G/A)* a						
GG (47)						
GA+AA (25)	0.51 (0.20–0.98)	0.04	0.70 (0.25–1.55)	0.40		
						
*XRCC3 (Thr241Met, C/T)* a						
CC (60)						
CT+TT (9)	0.75 (0.17–1.70)	0.54				
						
*Combined XPD and XRCC1*						
XPD AA and XRCC1 GG (40)						
Other (32)	0.41 (0.16–0.79)	0.006			0.50 (0.19–0.97)	0.04
						
*Total variant alleles in DNA repair genes*						
⩽3 (55)						
>3 (23)	0.51 (0.20–0.95)	0.03	0.77 (0.28–1.68)	0.53		
*Total variant alleles in NER genes* b						
⩽2 (50)						
>2 (28)	0.83 (0.47–1.37)	0.48				

TCC=transitional cell carcinoma; CR=complete response; PR=partial response; NC=no change; NER=nucleotide excision repair.

a For those polymorphisms with few homozygous variant alleles, only the combined results of the heterozygous and homozygous variant alleles are shown.

b NER genes include XPC, XPD, and XPG.

**Table 5:** Univariate and multivariate regression analyses for predicting cancer-specific survival in stage T3/T4 bladder cancer patients treated with chemoradiotherapy

	**Univariate model**		**Multivariate model one**		**Multivariate model two**	
**Variable (*n*)**	**Risk ratio (95% CI)**	***P*-value**	**Risk ratio (95% CI)**	***P*-value**	**Risk ratio *(95% CI)***	***P*-value**
*Age (years)*						
⩽68 (22)						
>68 (16)	1.07 (0.58–1.91)	0.81				
						
*Gender*						
Men (28)						
Women (10)	0.79 (0.31–1.53)	0.51				
						
*Tumour grade*						
2 (10)						
3 (28)	0.65 (0.37–1.20)	0.16				
						
*Histopathology*						
Pure TCC (32)						
Other elements (6)	2.10 (0.98–3.92)	0.06				
						
*Cisplatin dose (mg)*						
<235 (18)						
⩾235 (19)	1.58 (0.88–3.06)	0.13				
						
*Radiation dose (Gy)*						
<48.6 (13)						
⩾48.6 (25)	3.03 (1.32–13.01)	0.005	2.18 (0.92–9.44)	0.08	2.14 (0.92–9.20)	0.08
						
*Response*						
CR/PR (24)						
NC (14)	1.28 (0.69–2.28)	0.42				
						
*Salvage cystectomy*						
No (25)						
Yes (13)	0.54 (0.21–1.06)	0.08				
						
*XPC (Lys939Gln, A/C)*						
AA (10)						
AC (18)	0.94 (0.21–4.79)	0.94				
CC (6)	0.94 (0.12–5.69)	0.94				
AC+CC (24)	0.97 (0.50–2.11)	0.93				
						
*XPD (Lys751Gln, A/C)* a						
AA (27)						
AC+CC (8)	0.0004 (0.00–0.79)	0.02	0.0006 (not calculated)	0.08		
						
*XPG (Asp1104His, G/C)*						
GG (6)						
GC (21)	0.73 (0.16–5.02)	0.71				
CC (11)	1.23 (0.24–8.93)	0.81				
GC+CC (32)	0.96 (0.49–2.46)	0.92				
						
*XRCC1 (Arg399Gln, G/A)* a						
GG (26)						
GA+AA (9)	0.0004 (0.00–0.66)	0.005	0.0004 (not calculated)	0.02		
						
*XRCC3 (Thr241Met, C/T)* a						
CC (29)						
CT+TT (4)	0.92 (0.21–2.16)	0.87				
						
*Combined XPD and XRCC1*						
XPD AA and XRCC1 GG (20)						
Other (15)	0.0003 (0.00–0.45)	0.0002			0.0003 (0.00–0.53)	0.0008
						
*Total variant alleles in DNA repair genes*						
⩽3 (26)						
>3 (12)	0.39 (0.09–0.88)	0.02	0.87 (0.20–2.08)	0.79		
						
*Total variant alleles in NER genes* b						
⩽2 (22)						
>2 (16)	0.85 (0.44–1.52)	0.59				

TCC=transitional cell carcinoma; CR=complete response; PR=partial response; NC=no change; NER=nucleotide excision repair.

a For those polymorphisms with few homozygous variant alleles, only the combined results of the heterozygous and homozygous variant alleles are shown.

b NER genes include XPC, XPD, and XPG.

## References

[bib1] Bosken CH, Wei Q, Amos CI, Spitz MR (2002) An analysis of DNA repair as a determinant of survival in patients with non-small-cell lung cancer. J Natl Cancer Inst 94: 1091–1099 12122100 10.1093/jnci/94.14.1091

[bib2] Chakravarti A, Winter K, Wu CL, Kaufman D, Hammond E, Parliament M, Tester W, Hagan M, Grignon D, Heney N, Pollack A, Sandler H, Shipley W (2005) Expression of the epidermal growth factor receptor and Her-2 are predictors of favorable outcome and reduced complete response rates, respectively, in patients with muscle-invading bladder cancers treated by concurrent radiation and cisplatin-based chemotherapy: a report from the Radiation Therapy Oncology Group. Int J Radiat Oncol Biol Phys 62: 309–317 15890569 10.1016/j.ijrobp.2004.09.047

[bib3] Dunst J, Rodel C, Zietman A, Schrott KM, Sauer R, Shipley WU (2001) Bladder preservation in muscle-invasive bladder cancer by conservative surgery and radiochemotherapy. Semin Surg Oncol 20: 24–32 11291129 10.1002/ssu.1013

[bib4] Garcia del Muro X, Condom E, Vigues F, Castellsague X, Figures A, Munoz J, Sola J, Soler T, Capella G, Germa JR (2004) p53 and p21 Expression levels predict organ preservation and survival in invasive bladder carcinoma treated with a combined-modality approach. Cancer 100: 1859–1867 15112266 10.1002/cncr.20200

[bib5] Goode EL, Ulrich CM, Potter JD (2002) Polymorphisms in DNA repair genes and associations with cancer risk. Cancer Epidemiol Biomarkers Prev 11: 1513–1530 12496039

[bib6] Gu J, Zhao H, Dinney CP, Zhu Y, Leibovici D, Bermejo CE, Grossman HB, Wu X (2005) Nucleotide excision repair gene polymorphisms and recurrence after treatment for superficial bladder cancer. Clin Cancer Res 11: 1408–1415 15746040 10.1158/1078-0432.CCR-04-1101

[bib7] Gurubhagavatula S, Liu G, Park S, Zhou W, Su L, Wain JC, Lynch TJ, Neuberg DS, Christiani DC (2004) XPD and XRCC1 genetic polymorphisms are prognostic factors in advanced non-small-cell lung cancer patients treated with platinum chemotherapy. J Clin Oncol 22: 2594–2601 15173214 10.1200/JCO.2004.08.067

[bib8] Hoeijmakers JH (2001) Genome maintenance mechanisms for preventing cancer. Nature 411: 366–374 11357144 10.1038/35077232

[bib9] Hu JJ, Smith TR, Miller MS, Mohrenweiser HW, Golden A, Case LD (2001) Amino acid substitution variants of APE1 and XRCC1 genes associated with ionizing radiation sensitivity. Carcinogenesis 22: 917–922 11375899 10.1093/carcin/22.6.917

[bib10] Kachnic LA, Kaufman DS, Heney NM, Althausen AF, Griffin PP, Zietman AL, Shipley WU (1997) Bladder preservation by combined modality therapy for invasive bladder cancer. J Clin Oncol 15: 1022–1029 9060542 10.1200/JCO.1997.15.3.1022

[bib11] Khanna KK, Jackson SP (2001) DNA double-strand breaks: signaling, repair and the cancer connection. Nat Genet 27: 247–254 11242102 10.1038/85798

[bib12] Kumar R, Hoglund L, Zhao C, Forsti A, Snellman E, Hemminki K (2003) Single nucleotide polymorphisms in the XPG gene: determination of role in DNA repair and breast cancer risk. Int J Cancer 103: 671–675 12494477 10.1002/ijc.10870

[bib13] Lunn RM, Langlois RG, Hsieh LL, Thompson CL, Bell DA (1999) XRCC1 polymorphisms: effects on aflatoxin B1-DNA adducts and glycophorin A variant frequency. Cancer Res 59: 2557–2561 10363972

[bib14] Matsumoto H, Matsuyama H, Fukunaga K, Yoshihiro S, Wada T, Naito K (2004a) Allelic imbalance at 1p36 may predict prognosis of chemoradiation therapy for bladder preservation in patients with invasive bladder cancer. Br J Cancer 91: 1025–1031 15292937 10.1038/sj.bjc.6602073 PMC2747707

[bib15] Matsumoto H, Wada T, Fukunaga K, Yoshihiro S, Matsuyama H, Naito K (2004b) Bax to Bcl-2 ratio and Ki-67 index are useful predictors of neoadjuvant chemoradiation therapy in bladder cancer. Jpn J Clin Oncol 34: 124–130 15078907 10.1093/jjco/hyh026

[bib16] Matullo G, Peluso M, Polidoro S, Guarrera S, Munnia A, Krogh V, Masala G, Berrino F, Panico S, Tumino R, Vineis P, Palli D (2003) Combination of DNA repair gene single nucleotide polymorphisms and increased levels of DNA adducts in a population-based study. Cancer Epidemiol Biomarkers Prev 12: 674–677 12869411

[bib17] Mohrenweiser HW, Carrano AV, Fertitta A, Perry B, Thompson LH, Tucker JD, Weber CA (1989) Refined mapping of the three DNA repair genes, ERCC1, ERCC2, and XRCC1, on human chromosome 19. Cytogenet Cell Genet 52: 11–14 2558854 10.1159/000132829

[bib18] Paik ML, Scolieri MJ, Brown SL, Spirnak JP, Resnick MI (2000) Limitations of computerized tomography in staging invasive bladder cancer before radical cystectomy. J Urol 163: 1693–1696 10799162

[bib19] Park DJ, Stoehlmacher J, Zhang W, Tsao-Wei DD, Groshen S, Lenz HJ (2001) A Xeroderma pigmentosum group D gene polymorphism predicts clinical outcome to platinum-based chemotherapy in patients with advanced colorectal cancer. Cancer Res 61: 8654–8658 11751380

[bib20] Reed E, Ozols RF, Tarone R, Yuspa SH, Poirier MC (1987) Platinum-DNA adducts in leukocyte DNA correlate with disease response in ovarian cancer patients receiving platinum-based chemotherapy. Proc Natl Acad Sci USA 84: 5024–5028 3110781 10.1073/pnas.84.14.5024 PMC305239

[bib21] Rödel C, Grabenbauer GG, Kuhn R, Papadopoulos T, Dunst J, Meyer M, Schrott KM, Sauer R (2002) Combined-modality treatment and selective organ preservation in invasive bladder cancer: long-term results. J Clin Oncol 20: 3061–3071 12118019 10.1200/JCO.2002.11.027

[bib22] Ryu JS, Hong YC, Han HS, Lee JE, Kim S, Park YM, Kim YC, Hwang TS (2004) Association between polymorphisms of ERCC1 and XPD and survival in non-small-cell lung cancer patients treated with cisplatin combination chemotherapy. Lung Cancer 44: 311–316 15140544 10.1016/j.lungcan.2003.11.019

[bib23] Sak SC, Harnden P, Johnston CF, Paul AB, Kiltie AE (2005) APE1 and XRCC1 protein expression levels predict cancer-specific survival following radical radiotherapy in bladder cancer. Clin Cancer Res 11: 6205–6211 16144922 10.1158/1078-0432.CCR-05-0045

[bib24] Sakano S, Berggren P, Kumar R, Steineck G, Adolfsson J, Onelöv E, Hemminki K, Larsson P (2003) Clinical course of bladder neoplasms and single nucleotide polymorphisms in the CDKN2A gene. Int J Cancer 104: 98–103 12532425 10.1002/ijc.10919

[bib25] Sakano S, Kumar R, Larsson P, Onelöv E, Adolfsson J, Steineck G, Hemminki K (2006) A single-nucleotide polymorphism in the XPG gene, and tumour stage, grade, and clinical course in patients with nonmuscle-invasive neoplasms of the urinary bladder. BJU Int 97: 847–851 16536785 10.1111/j.1464-410X.2005.05994.x

[bib26] Sanyal S, Festa F, Sakano S, Zhang Z, Steineck G, Norming U, Wijkstrom H, Larsson P, Kumar R, Hemminki K (2004) Polymorphisms in DNA repair and metabolic genes in bladder cancer. Carcinogenesis 25: 729–734 14688016 10.1093/carcin/bgh058

[bib27] Schilsky RL (1992) Biochemical pharmacology of chemotherapeutic drugs used as radiation enhancers. Semin Oncol 19 (Suppl 11): 2–7 1509277

[bib28] Shipley WU, Kaufman DS, Tester WJ, Pilepich MV, Sandler HM, Radiation Therapy Oncology Group (2003) Overview of bladder cancer trials in the Radiation Therapy Oncology Group. Cancer 97: 2115–2119 12673704 10.1002/cncr.11282

[bib29] Shipley WU, Prout Jr GR, Einstein AB, Coombs LJ, Wajsman Z, Soloway MS, Englander L, Barton BA, Hafermann MD (1987) Treatment of invasive bladder cancer by cisplatin and radiation in patients unsuited for surgery. JAMA 258: 931–935 3613023

[bib30] Shipley WU, Winter KA, Kaufman DS, Lee WR, Heney NM, Tester WR, Donnelly BJ, Venner PM, Perez CA, Murray KJ, Doggett RS, True LD (1998) Phase III trial of neoadjuvant chemotherapy in patients with invasive bladder cancer treated with selective bladder preservation by combined radiation therapy and chemotherapy: initial results of Radiation Therapy Oncology Group 89-03. J Clin Oncol 16: 3576–3583 9817278 10.1200/JCO.1998.16.11.3576

[bib31] Spitz MR, Wu X, Wang Y, Wang LE, Shete S, Amos CI, Guo Z, Lei L, Mohrenweiser H, Wei Q (2001) Modulation of nucleotide excision repair capacity by XPD polymorphisms in lung cancer patients. Cancer Res 61: 1354–1357 11245433

[bib32] Stoehlmacher J, Park DJ, Zhang W, Yang D, Groshen S, Zahedy S, Lenz HJ (2004) A multivariate analysis of genomic polymorphisms: prediction of clinical outcome to 5-FU/oxaliplatin combination chemotherapy in refractory colorectal cancer. Br J Cancer 91: 344–354 15213713 10.1038/sj.bjc.6601975 PMC2409815

[bib33] Tester W, Caplan R, Heaney J, Venner P, Whittington R, Byhardt R, True L, Shipley W (1996) Neoadjuvant combined modality program with selective organ preservation for invasive bladder cancer: results of Radiation Therapy Oncology Group phase II trial 8802. J Clin Oncol 14: 119–126 8558186 10.1200/JCO.1996.14.1.119

[bib34] Thompson LH, West MG (2000) XRCC1 keeps DNA from getting stranded. Mutat Res 459: 1–18 10677679 10.1016/s0921-8777(99)00058-0

[bib35] Vodicka P, Kumar R, Stetina R, Sanyal S, Soucek P, Haufroid V, Dusinska M, Kuricova M, Zamecnikova M, Musak L, Buchancova J, Norppa H, Hirvonen A, Vodickova L, Naccarati A, Matousu Z, Hemminki K (2004) Genetic polymorphisms in DNA repair genes and possible links with DNA repair rates, chromosomal aberrations and single-strand breaks in DNA. Carcinogenesis 25: 757–763 14729591 10.1093/carcin/bgh064

[bib36] Yoon SM, Hong YC, Park HJ, Lee JE, Kim SY, Kim JH, Lee SW, Park SY, Lee JS, Choi EK (2005) The polymorphism and haplotypes of XRCC1 and survival of non-small-cell lung cancer after radiotherapy. Int J Radiat Oncol Biol Phys 63: 885–891 16199318 10.1016/j.ijrobp.2005.07.951

[bib37] Zhou W, Gurubhagavatula S, Liu G, Park S, Neuberg DS, Wain JC, Lynch TJ, Su L, Christiani DC (2004) Excision repair cross-complementation group 1 polymorphism predicts overall survival in advanced non-small cell lung cancer patients treated with platinum-based chemotherapy. Clin Cancer Res 10: 4939–4943 15297394 10.1158/1078-0432.CCR-04-0247

